# Supplementary material for: Cardiac function assessment with deep-learning-based automatic segmentation of free-running four-dimensional whole-heart cardiovascular magnetic resonance
Source: J Cardiovasc Magn Reson. 2025 Dec 24;28(1):102677. doi: 10.1016/j.jocmr.2025.102677 (PMC13112478; doi:10.1016/j.jocmr.2025.102677)
Supplement: Supplementary file 1 — Supplementary material [file mmc1.pdf]

Supplementary Material

Supplementary Movie M1: A) 3D visualization of the heart region throughout the cardiac cycle, showing three orthogonal planes and dynamic surface meshes of the left ventricular blood pool (LVB, red), left ventricular myocardium (LVM, green), and right ventricular blood pool (RVB, purple). B) Free-running (FR) images are displayed in pseudo two-chamber, pseudo four-chamber, and short-axis views (left to right), corresponding to the orthogonal planes used in the semi-automatic segmentation that served as ground truth for deep learning training. Segmented structures are color-coded as in the 3D view. For both A and B, the frame rate is 50 ms, matching the temporal resolution of the reconstructed images. Note that while the slice orientations are consistent between A and B, the slice positions are not spatially matched. Data shown is from a healthy volunteer in their 60s, acquired at 3T (dataset D_2).

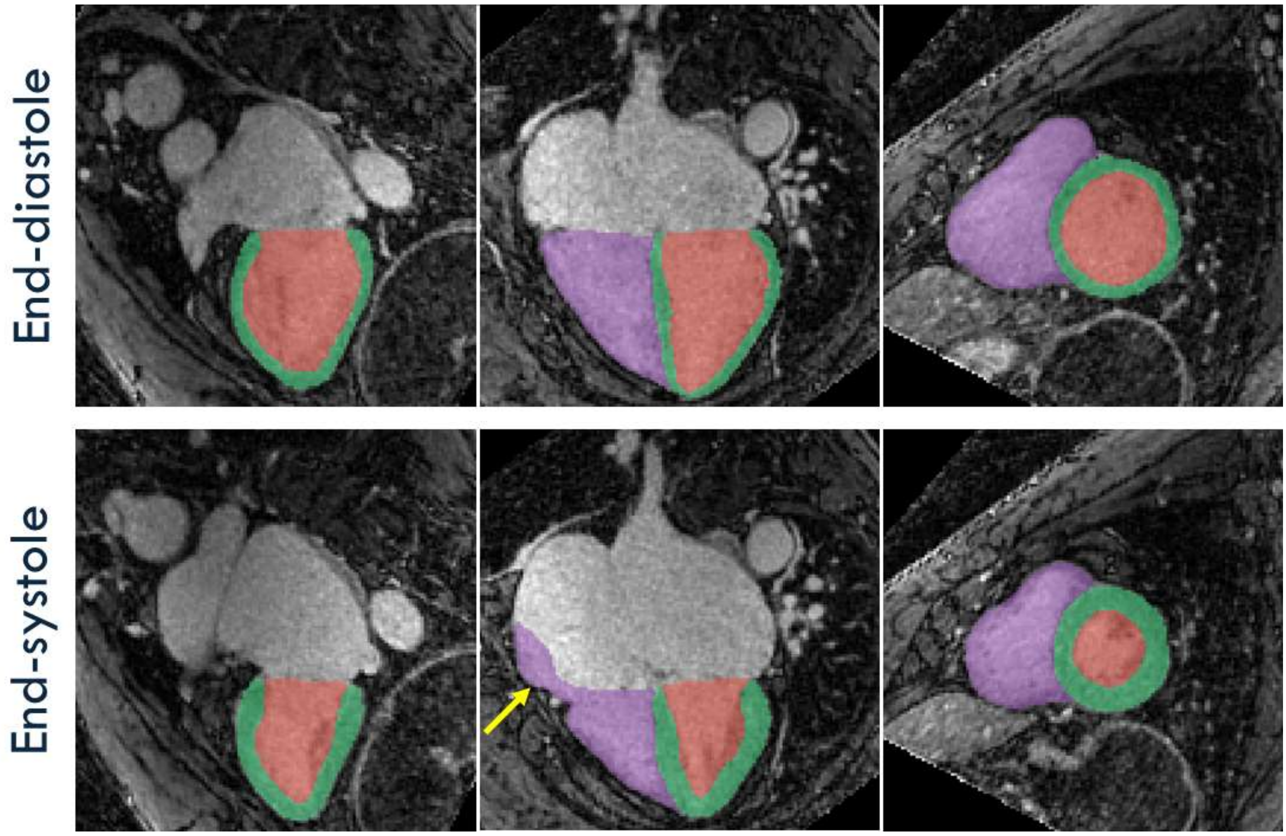

Supplementary Figure S1: Segmentation mismatch in the case of non-standard anatomy. DL-based automatic segmentation (FR_A) of spatially isotropic 4D free-running (FR) images at end-diastole (top) and end-systole (bottom) for a patient in their 70s with HFpEF, acquired at 3T (dataset D_2). The subject presents with marked bi-atrial dilation. FR images are shown in pseudo two-chamber, pseudo four-chamber, and short-axis view (left to right), corresponding to the orthogonal planes used during semi-automatic ground truth segmentation. The right ventricle blood pool (purple), the left ventricle myocardium (green), and left ventricle blood pool (red) are highlighted. The automatic segmentation shows an overextension at end-systole only of the right ventricle contour into the right atrium (arrow), illustrating a common challenge in RV delineation under pathological remodeling.

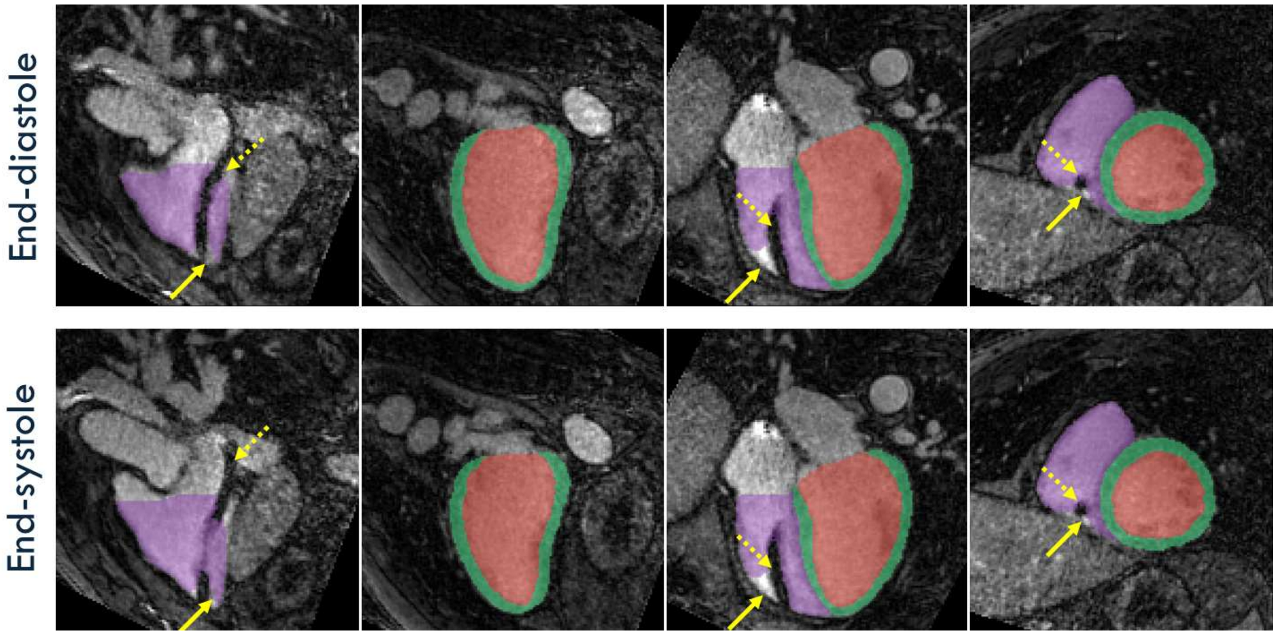

Supplementary Figure S2: Partial segmentation failure in the presence of a device artifact. DL-based automatic segmentation (FR_A) of spatially isotropic 4D free-running (FR) images at end-diastole (top) and end-systole (bottom) for a patient in their 60s with HFrEF, acquired at 3T (dataset D_2), showing an imaging artifact (dashed arrows) due to a implantable cardioverter defibrillator (ICD) lead (Acticor 7 VR-T, Biotronik, Berlin, Germany) in the right ventricle (RV). FR images are shown in RV pseudo two-chamber, LV pseudo two-chamber, pseudo four-chamber, and short-axis view (left to right). The DL model segmented the RV blood pool (purple), left ventricle myocardium (green), and left ventricle blood pool (red) accurately across the heart, except in the immediate vicinity of the ICD artifact (dashed arrows), where segmentation performance was reduced (solid arrows). This case highlights the model's general robustness, along with challenges posed by uncommon artifacts.

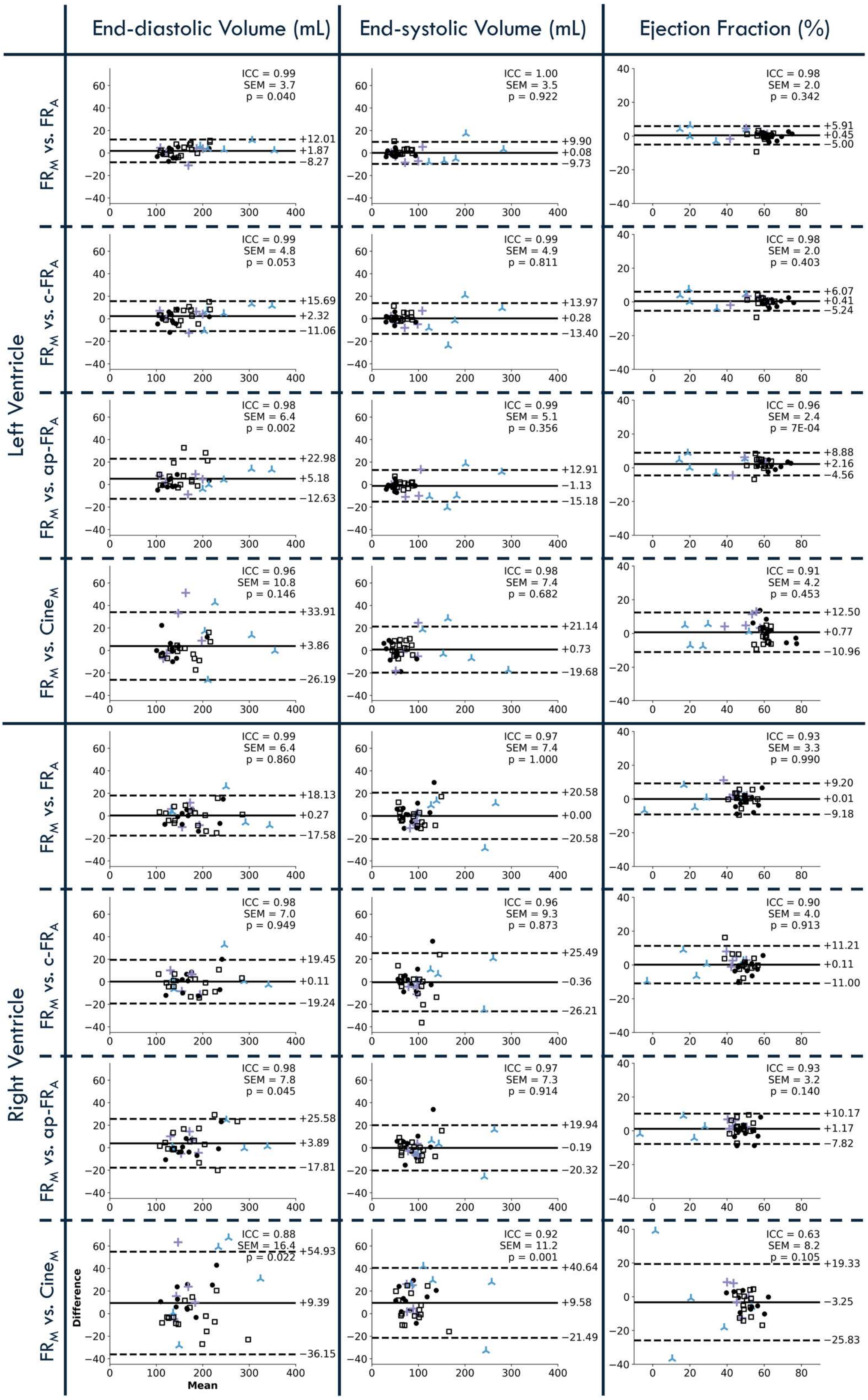

Supplementary Figure S3. Clinical metrics comparison through volumetric and functional agreement for all segmentation strategies. Bland-Altman analysis comparing semi-automatic segmentation (FR_M) with four other methods: standard deep learning-based segmentation (FR_A), native canonical space model ($c\text{-}FR_A$), all cardiac phases ($ap\text{-}FR_A$), and manual segmentation of cine images ($Cine_M$) for end-diastolic volume, end-systolic volume, and ejection fraction in both the left and right ventricles. Healthy volunteers are represented by black dots (square markers for dataset D_1, circle markers for dataset D_2), patients with HFrEF by blue crosses, and those with HFpEF by purple crosses.

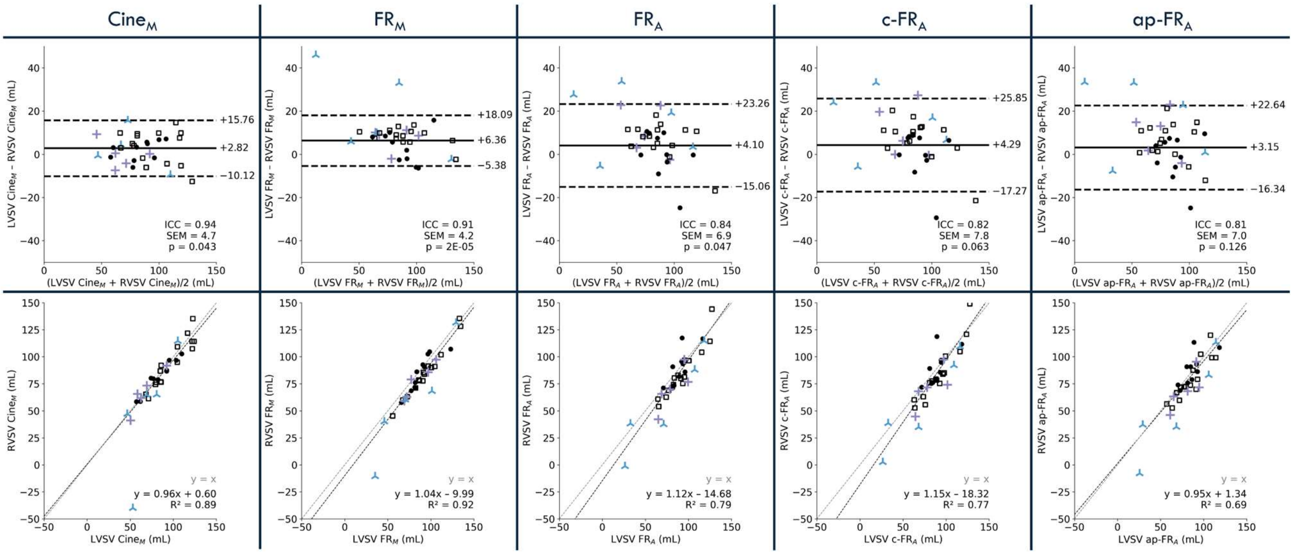

Supplementary Figure S4: LV–RV stroke volume agreement. Bland-Altman analysis (top) and correlation plots (bottom) comparing stroke volumes of the right ventricle (RVSV) and left ventricle (LVSV), computed from manual segmentation of cine images ($Cine_M$) and from semi-automatic segmentation of 4D FR images (FR_M), and automatic deep learning-based segmentations (FR_A, c-FR_A, and ap-FR_A). Healthy volunteers are represented by black dots (square markers for dataset D_1, circle markers for dataset D_2), patients with HFrEF by blue crosses, and those with HFpEF by purple crosses.
